# G9a promotes immune suppression by targeting the Fbxw7/Notch pathway in glioma stem cells

**DOI:** 10.1111/cns.14191

**Published:** 2023-03-27

**Authors:** Yufei Cao, Bin Liu, Lize Cai, Yanyan Li, Yulun Huang, Youxin Zhou, Xingjian Sun, Wei Yang, Ting Sun

**Affiliations:** ^1^ Neurosurgery and Brain and Nerve Research Laboratory The First Affiliated Hospital of Soochow University Suzhou Jiangsu China; ^2^ Department of Neurosurgery Qinghai Provincial People's Hospital Xining Qinghai China; ^3^ Department of Neurosurgery Dushu Lake Hospital Affiliated of Soochow University Suzhou Jiangsu China; ^4^ State Key Laboratory of Radiation Medicine and Protection School of Radiation Medicine and Protection and Collaborative Innovation Center of Radiation Medicine of Jiangsu Higher Education Institutions Soochow University Suzhou Jiangsu China

**Keywords:** Fbxw7, G9a, glioma stem cells, immune suppression, notch

## Abstract

**Aim:**

Immunotherapy for glioblastoma multiforme (GBM) is limited because of a strongly immunosuppressive tumor microenvironment (TME). Remodeling the immune TME is an effective strategy to eliminate GBM immunotherapy resistance. Glioma stem cells (GSCs) are inherently resistant to chemotherapy and radiotherapy and involved in immune evasion mechanism. This study aimed to investigate the effects of histone methyltransferases 2 (EHMT2 or G9a) on immunosuppressive TME and whether this effect was related to changes on cell stemness.

**Methods:**

Tumor‐infiltrating immune cells were analyzed by flow cytometry and immunohistochemistry in orthotopic implanted glioma mice model. The gene expressions were measured by RT‐qPCR, western blot, immunofluorescence, and flow cytometry. Cell viability was detected by CCK‐8, and cell apoptosis and cytotoxicity were detected by flow cytometry. The interaction of G9a and F‐box and WD repeat domain containing 7 (Fbxw7) promotor was verified by dual‐luciferase reporter assay and chromatin immunoprecipitation.

**Results:**

Downregulation of G9a retarded tumor growth and extended survival in an immunocompetent glioma mouse model, promoted the filtration of IFN‐γ + CD4+ and CD8+ T lymphocytes, and suppressed the filtration of PD‐1+ CD4+ and CD8+ T lymphocytes, myeloid‐derived suppressor cells (MDSCs) and M2‐like macrophages in TME. G9a inhibition decreased PD‐L1 and increased MHC‐I expressions by inactivating Notch pathway companying stemness decrease in GSCs. Mechanistically, G9a bound to Fbxw7, a Notch suppressor, to inhibit gene transcription through H3K9me2 of Fbxw7 promotor.

**Conclusion:**

G9a promotes stemness characteristics through binding Fbxw7 promotor to inhibit Fbxw7 transcription in GSCs, forming an immunosuppressive TME, which provides novel treatment strategies for targeting GSCs in antitumor immunotherapy.

## Graphical abstract

G9a promotes histone H3 lysine 9 methylation on Fbxw7 promotor, resulting in the inhibition of Fbxw7 transcription. Downregulated Fbxw7 activates Notch pathway in glioma stem cells to suppress immune response by regulating the expression of immune‐associated molecules.

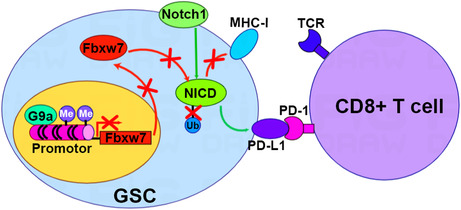

## 1 INTRODUCTION

Glioblastoma multiforme (GBM) is the most lethal primary tumor in the central nervous system. Although precise surgical resection in combination of radiotherapy and chemotherapy, most tumors inevitably recur, and the median survival time is less than 15 months.^1^ Glioma stem cells (GSCs), a small subpopulation of GBM cells, have the capability to self‐renew, proliferate, and multiple differentiation. GSCs are inherently resistant to chemotherapy and radiotherapy, causing invasion, and recurrence.^2^ Several strategies including in facilitating an inflammatory tumor microenvironment (TME), targeting stemness markers, and changing tumor characteristics by epigenetic modification make GSCs more susceptible to therapy in GBM.^3^

Notch signaling pathway plays an important role in promoting tumor dormancy escape, recurrence, and progression after conventional therapy.^4^ Notch intracellular domain (NICD), the active intracellular form of Notch after ligand binding, in the nucleus of tumor cells has been demonstrated using immunohistochemistry in human glioma samples.^5^ Notch1 expression is higher in high‐grade glioma compared to low‐grade glioma.^6^ Previous study has shown that activated Notch1 maintained a stemness phenotype in GSCs, and its downstream targets were highly upregulated in GSCs.^7^ Notch inhibitor decreased population growth, clonogenicity, and expressions of GSCs markers in GBM‐derived neurospheres,^8^ facilitating conventional therapy.

A large number of immunosuppressive cells are involved in GBM microenvironment, and GBM cells generate immunosuppressive factors and highly express immune checkpoint ligand to inhibit local immune response.^9^ Activated Notch signal in GSCs regulates molecular adaptation to the local immunosuppressive TME.^10^ Single‐cell RNA sequencing found that Notch signaling regulated tumor immune microenvironment, and Notch blockade delayed tumor recurrence.^11^ Targeting Notch pathway, making tumor in an inflammatory TME, may be an effective strategy to sensitize immunotherapy.

The epigenetic modifiers have important functions on regulating gene expression and remodeling chromatin through DNA methylation or histone modifications.^12^ Histone methyltransferases 2 (EHMT2 or G9a) as a permanent epigenetic marker predominantly governs histone H3 lysine 9 methylation (H3K9me). Studies have identified that G9a was correlated with malignancies, and G9a inhibitor decreased glioma cell viability.^13^, ^14^ G9a upregulation and concomitant H3K9me modification promoted transcriptional suppression of multiple genes. Lower H3K9me2 levels at the promoters of autophagy and differentiation‐related genes were identified in differentiated cells than in GSCs. G9a inhibitors BIX01294 treatment upregulated the expressions of these genes in tumor spheres,^15^ suggesting that G9a regulates cell stemness in GSCs. However, whether G9a is involved in the epigenetic regulation of Notch gene transcription in GSCs remains unclear.

Herein, we investigated the mechanism in which G9a remodeled tumor immune microenvironment by regulating stem cell signaling pathway. Our data showed that G9a promoted the expression of Notch signaling, and further demonstrated that G9a‐mediated epigenetic silencing of F‐box and WD repeat domain containing 7 (Fbxw7), a known Notch suppressor, elevated the expression of Notch1, which influenced the expressions of stem cell markers and immune‐associated molecules in GBM progression.

## 2 MATERIALS AND METHODS

### 2.1 Cell culture

Human GSCs line 51A was from GBM patient as described previously.^16^ GSCs were cultured in serum‐free DMEM/F12 medium supplied with 2% B27, 20 ng/mL epidermal growth factor (EGF), and 20 ng/mL basic fibroblast growth factor (bFGF). 51A GSCs were cultured in high‐glucose DMEM with 10% FBS to produce non‐GSCs for 1 month. Human glioma cell line SHG140 was from primary culture of patient's glioma tissue in the First Affiliated Hospital of Soochow University.^17^ Human glioma cell lines U251 and U87 were purchased from the Shanghai Institutes for Biological Sciences. Glioma cells were cultured in the medium of GSCs for 1 month, then CD133+ cells were sorted using CD133 MicroBead (Miltenyi Biotec) according to producer's direction, and defined as GSCs. The murine glioma cell line GL261 was obtained from American Type Culture Collection (ATCC), and cultured in high‐glucose DMEM with 10% FBS. All cells were used after identification as previously described.^16^, ^17^

### 2.2 Animal experiments in vivo

Male C57BL/6J and nude mice 18–20 g were fed in specific pathogen‐free facilities at the Soochow University Animal Center. Experimental protocol was approved by the Medical Ethics Committee of the First Affiliated Hospital of Soochow University.

**FIGURE 1:**
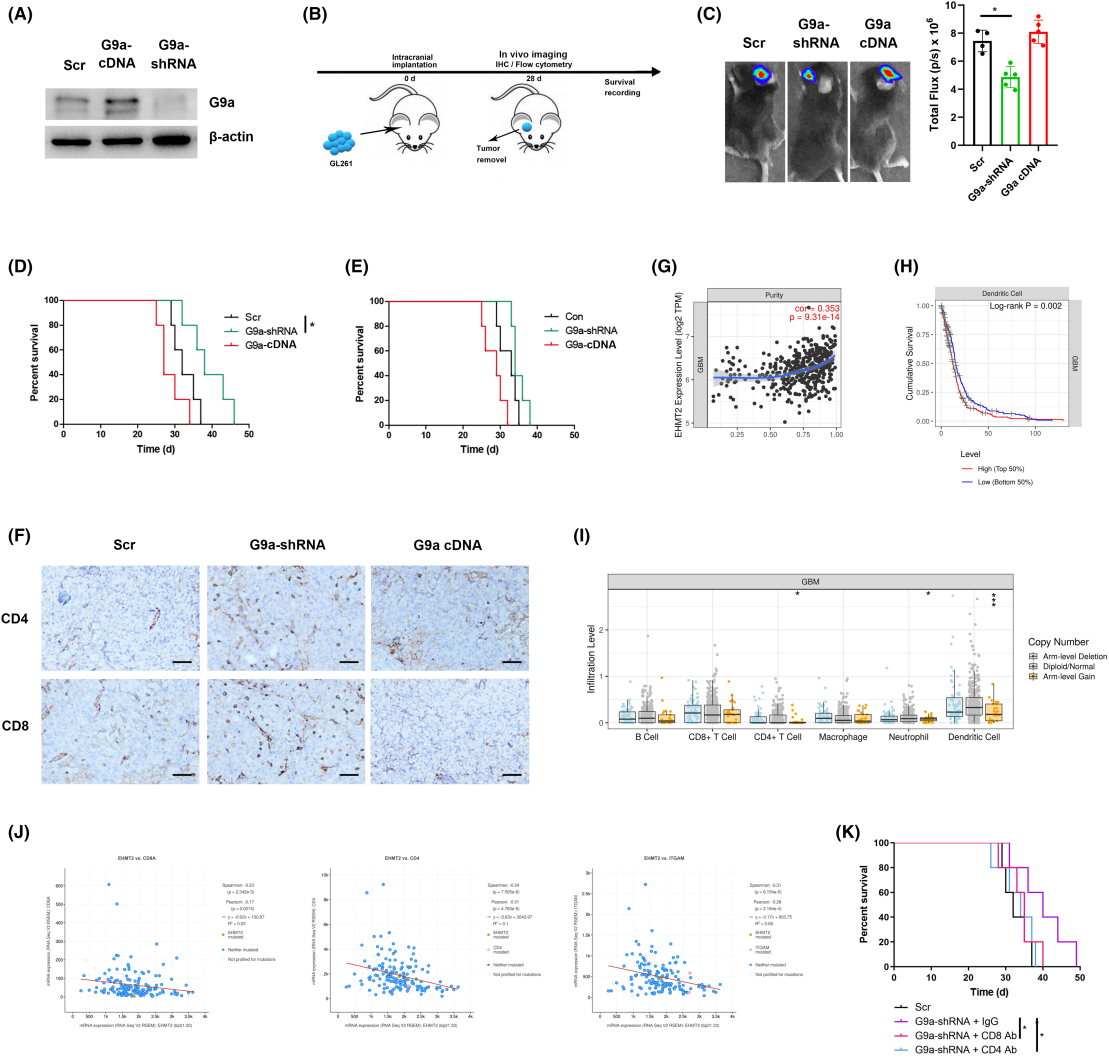
G9a expression in glioma cells decreased survival time and promoted immune suppression in vivo. (A) G9a expressions were detected in G9a‐shRNA or G9a cDNA‐transfected GL261 cells using western blot. (B) Schema of the C57BL/6J mouse tumor model was shown for survival recording, IHC, and flow cytometry assay. (C) GL261 cells transfected with scramble (Scr), G9a‐shRNA, or G9a cDNA lentivirus were intracranially implanted into C57BL/6J mice, then tumor size were detected using in vivo imaging. (D) The survival of C57BL/6J mice was recorded. (E) Mice survival was shown in tumor‐bearing nude mice after implantation of GL261 cells. (F) Representative images of CD4 and CD8‐positive cells were shown in allografts of C57BL/6J mice from GL261 cells by IHC staining. Images were captured with a light microscope (×200). Scale bars correspond to 50 μm. Correlations between G9a, encoded by the euchromatic histone lysine N‐methyltransferase 2 (EHMT2) gene, and tumor purity (G), immune cells‐associated survival (H), and immune cells infiltration (I) were analyzed by TIMER database. (J) The correlation analysis of G9a and immune cell markers from TCGA dataset was shown. (K) CD4 or CD8+ T cells were depleted using anti‐CD4 or ant‐CD8α antibody in tumor‐bearing C57BL/6J mice implanted with G9a‐shRNA‐transfected GL261 cells, and the survival of mice was recorded. *n* = 5, *p < 0.05.

For tumor challenge experiments, C57BL/6J mice or nude mice were intracranially injected with 1 × 10^5^ GL261 cells in 5 μL into the frontal lobe of cerebrum to establish intracranial allografts. All mice were sacrificed when they experienced suffering symptoms including inactivity, feeding interfere, and severe weight loss, and survival was recorded. In vivo imaging system (IVIS) 50 system was used to quantify the size of grafts at day 28 after C57BL/6J mice were implanted with GL261 cells carrying luciferase lentivirus. Bioluminescence imaging signal was reported as average flux. Intracranial grafts in mice were surgically resected to perform IHC or flow cytometry on the same day (Figure 1B). For CD4 or CD8 T cell deletion, 20 μg of IgG, CD4, or CD8 antibody in 100 μL was i.p. administered three times per week.

### 2.3 Cell transfection and stable cell lines generation

Cells were transfected with G9a‐shRNA and Fbxw7‐shRNA for gene knockdown. Plasmids carrying human G9a wild‐type cDNA or SET site (913–1193 aa)‐deleted G9a cDNA^18^ were constructed and transfected into GSCs for gene overexpression, respectively. For stable cell line construction, lentivirus carrying cDNA or shRNA targeting G9a was generated and transfected according to producer's direction. All lentivirus and shRNA were constructed by genepharma Co., Ltd. Targeting shRNA sequences for G9a is 5’‐CACACATTCCTGACCAGAGAT‐3′ and shRNA for Fbxw7 is 5’‐CCAGTCGTTAACAAGTGGAAT‐3′.

### 2.4 Immunohistochemical (IHC) staining

The tissues were fixed in formalin, embedded in paraffin, and sectioned at 4 μm. The slides were blocked with 5% BSA, incubated with primary antibodies including anti‐CD4 and anti‐CD8 (Abcam) at 4°C overnight and horseradish peroxidase‐conjugated goat anti‐rabbit IgG at room temperature for 30 min. The reactions were visualized by a diaminobenzidine (DAB) substrate, and photographs were captured under light microscope.

### 2.5 Flow cytometry

Tumor tissues from C57BL/6J mice were collected and dissociated into single‐cell suspension by filtering with a 70‐μm mesh cell strainer. Red blood cells were lysed in ACK lysing buffer. Cells were resuspended in Percoll gradients and centrifuged at 400 *g* for 20 min to isolate lymphocytes. PMA, ionophore, and protein transport inhibitor were added into cell suspension for 5 h incubation. For lymphocyte subpopulations. cells were stained for analysis of surface markers including anti‐CD3 eFluor™ 450 (eBioscience), anti‐CD4 FITC (eBioscience), anti‐CD8 APC (eBioscience), and anti‐PD‐1 PE‐Cy7 (eBioscience), then fixed and permeabilized for anti‐IFN‐γ PE (eBioscience) measurement. For myeloid‐derived suppressor cells (MDSCs) and M2‐like macrophage subpopulations, cells were stained with anti‐CD11b eFluor™ 450 (eBioscience), anti‐Ly6G PE‐Cy7 (eBioscience), anti‐Ly6C PE (eBioscience), anti‐F4/80 APC (eBioscience), and anti‐CD206 FITC (eBioscience). Dead cells were stained with 7‐AAD.

Cultured cells in vitro were stained with PE‐conjugated HLA‐ABC, H‐2Kb, and PD‐L1 (eBioscience) monoclonal antibodies in the dark at 4°C for 1 h, then analyzed using a flow cytometer (BD Bioscience).

### 2.6 Reverse transcription quantitative polymerase chain reaction (RT‐qPCR)

Total RNA was isolated by TRIzol reagent (Invitrogen) and reverse‐transcribed to cDNA with a 1st Strand cDNA Synthesis Kit (Thermo Scientific). PCR was performed with SYBR Green PCR Master Mix (Takara). All the steps were done according to the manufacturer's instructions. PCR primers are the following: for G9a, forward: 5′‐AGGCACCCAAGATTGACC‐3′, reverse: 5′‐GTCTCCCGCTTGAGGATG‐3′; for Fbxw7, forward: 5’‐GGCGCCGCGGCTCTTTTCTA‐3′, reverse: 5’‐GCTGCCCACAGAGAGCAGTTCC‐3′.

### 2.7 Western blot

Total protein was extracted from lysis buffer (Beyotime Biotechnology) containing phosphatase inhibitor cocktail on ice. The protein content was determined, then 20 μg of total protein was separated by SDS‐PAGE gel and transferred onto PVDF membrane (Millipore). The membrane was blocked with 5% BSA for 2 h, then incubated with primary antibodies overnight at 4°C. After incubated with HRP‐coupled secondary antibodies for 2 h at room temperature, the protein was analyzed by an enhanced chemiluminescence kit (Beyotime) and detected by chemiluminescence system (Bio‐Rad).

### 2.8 Immunofluorescence staining

Cells were fixed by 4% paraformaldehyde for 15 min, permeabilized by 0.1% Triton X‐100 for 10 min, then blocked with 1% BSA for 1 h. Primary antibodies including anti‐SOX2 (Cell Signaling) and anti‐Oct4 (Cell Signaling) were used for immunostaining at 4°C overnight, then Alexa Fluor 488 or Alexa Fluor 555‐conjugated secondary IgG antibody (Invitrogen) was added for reaction. The nuclei were stained with DAPI, and Images were captured by a fluorescence microscope.

### 2.9 Dual‐luciferase reporter assay

The binding of G9a and Fbxw7 promotor was validated using a dual‐luciferase reporter assay. The Fbxw7 promotor was synthesized and inserted into pGL3 luciferase vectors. The wild‐type and SET deleted sequences for G9a were constructed by genepharma Co., Ltd. Fbxw7 promotor reporter plasmids were transfected into GSCs with G9a shRNA, wild‐type or SET‐deleted plasmid. Luciferase activity was measured 48 h after transfection using a dual‐luciferase reporter gene assay system (Promega).

### 2.10 Chromatin immunoprecipitation (ChIP)

ChIP experiment was performed using Chromatin IP Kit (Cell Signaling) according to manufacturer's protocol. In brief, GSCs were added with 37% formaldehyde for 10 min, then DNA was processed to the length of 150–900 bp by nuclease digestion and sonication. Chromatin fragment of 10 μg was mixed with G9a (Cell Signaling) or H3K9me2 (Cell Signaling) antibody at 4°C overnight. Protein G magnetic beads of 30 μL were added and incubated for 2 h at 4°C. Chromatin was eluted from beads and determined using qPCR reactions. ChIP primers are the following: Forward: 5’‐CCGGGAGAAGTGGCCCTGGA‐3′, Reverse: 5’‐GAAGCGGTGCTCGTGTCGCT‐3′.

### 2.11 Cell viability analysis

Cell viability was assessed using a CCK‐8 assay according to the manufacturer's instructions. Briefly, 5000 cells were seeded in a 96‐well plate and treated with UNC0642 at the indicated concentration. CCK‐8 was added and optical density at 492 nm was read using a microplate reader.

### 2.12 Apoptosis analysis

Cells were treated with 20 μM UNC0642 for 24 h or transfected with G9a‐shRNA for 48 h, then stained with PE Annexin V Apoptosis Detection Kit with 7‐AAD (Biolegend) according to the manufacturer's instructions. Apoptotic cells were analyzed by flow cytometry.

### 2.13 Spheroid formation assay

The primary spheres were dissociated mechanically into single cells and resuspended in DMEM/F12 medium with EGF, bFGF, and B27. Tumorspheres were observed and counted under an inverted microscope 5 days later.

### 2.14 T‐cell cytotoxicity assay

Human peripheral blood was collected in lithium heparin tubes, and peripheral blood mononuclear cells (PBMCs) were separated by density‐gradient centrifugation using Lymphocyte Separation Medium (Corning). Enriched CD8+ T cells by EasySep™ Human CD8+ T Cell Enrichment Kit (StemCell Technologies) were activated with anti‐CD3 (1 μg/mL) and anti‐CD28 (5 μg/mL) antibodies (eBioscience) for 5 days. Murine CD8+ T cells were isolated from spleen of C57BL/6J mice. Tumor cells were co‐cultured with CD8+ T cells at indicated ratio, then cytotoxicity was detected using flow cytometry. Tumor cells were prelabeled by CFSE, dead cells were stained using PI, and the double‐positive cells of CFSE and PI were considered as dead tumor cells.

### 2.15 Statistical analysis

Statistical analyses were performed using the Graphpad Prism 8.0 software. All data are presented as the mean ± standard deviation. Unpaired two‐tailed Student's *t*‐test was performed to compare two groups, and one‐way analysis of variance (ANOVA) with Tukey's post hoc test was performed among multiple groups. Prior to statistical analyses, the datasets for each group were tested for normality of distribution using the Kolmogorov–Smirnov test. Results with *p* values of <0.05 or <0.01 were set to be significant.

## 3 RESULTS

### 3.1 G9a function in tumor growth promotion depends on the immune system

To analyze the role of G9a in tumor growth and survival, we stably transfected lentivirus vector carrying shRNA to knockdown G9a or cDNA to overexpress G9a in mouse GL261 cells (Figure 1A). G9a knockdown notably retarded tumor growth (Figure 1B,C) and extended survival time in immunocompetent C57BL/6 mice (Figure 1D). To examine whether the retardation on tumor growth and the extension on survival of tumor‐bearing mice by G9a knockdown are associated with host immune response, nude mice, deficient in T cells were inoculated with GL261 cells. Compared with immunocompetent mice, the difference in survival time was decreased in nude mice with GL261 cells transfected with scramble and G9a‐shRNA (Figure 1E). IHC also showed the increased infiltration of CD4+ and CD8+ T cells in allografts of C57BL/6 mice with G9a‐shRNA‐transfected GL261 cells compared with scramble sequence transfection (Figure 1F). Further assessment using TIMER (Tumor Immune Estimation Resource) database showed that G9a expression was positively associated with GBM purity (Figure 1G). The survival of GBM patients with G9a high expression exhibited significantly dendritic cells (DCs)‐associated poor clinical outcome (Figure 1H), and G9a expression was associated with DCs infiltration level (Figure 1I). Moreover, the correlation analysis from TCGA dataset showed that G9a displayed a negative correlation with the expressions of immune cell markers including CD4, CD8α (CD8A), and CD11C (ITGAX) (Figure 1J). To further determine how G9a affected the antitumor immunity, IgG, CD4 or CD8 antibody was injected into C57BL/6 mice with allografts to deplete CD4+ or CD8+ T cells. CD8+ T cell depletion significantly shortened survival time of C57BL/6 mice with G9a‐shRNA‐transfected GL261 cells (Figure 1K). The results suggested that G9a promoted tumor growth in vivo partly through the suppression on antitumor immune response.

### 3.2 G9a inhibited local antitumor immunity and modulated immune cell subpopulations

**FIGURE 2:**
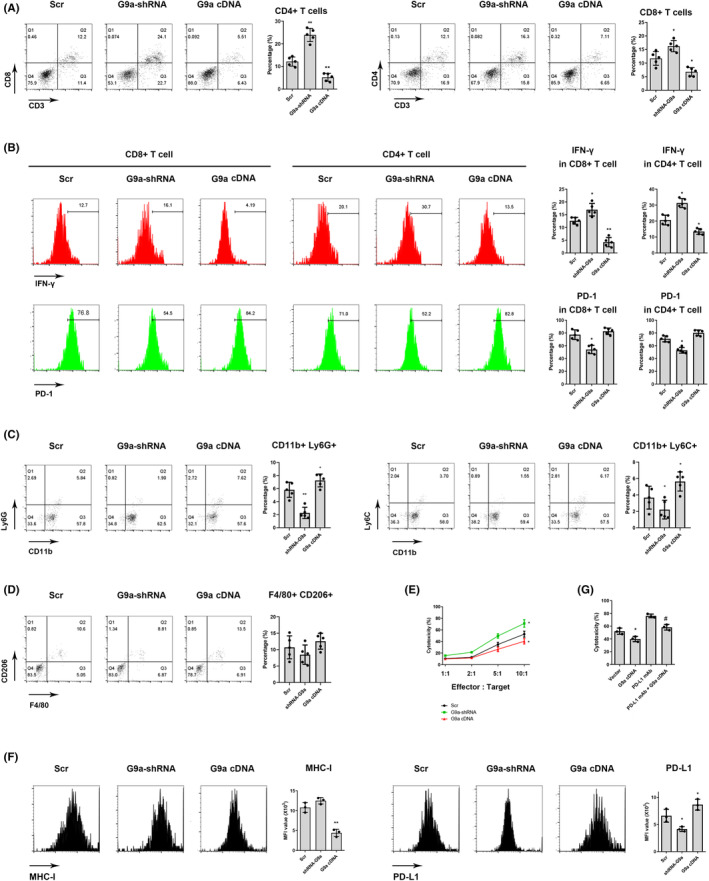
G9a inhibited lymphocytes infiltration in glioma tissue and regulated immune molecules expression in glioma cells. (A) Tumor tissue from GL261 cell‐bearing mice was removed and dissociated into single‐cell suspension (schedule shown in Figure 1B), then CD4+ and CD8+ T cells were analyzed using flow cytometry. *n* = 5. (B) IFN‐γ releasing and PD‐1‐expressing immune cells were showed. (C) The percentage of G‐MDSCs and M‐MDSCs was analyzed using flow cytometry. (D) M2‐like macrophages were in all macrophage subpopulations were showed. (E) CD8+ T cells were purified from C57BL/6J mice spleen, then added into G9a‐shRNA or G9a cDNA‐transfected GL261 cells at indicated ratio of effector: target cells for 24 h co‐culture. GL261 cells were labeled by CFSE, and double‐positive cells of CFSE and PI staining were evaluated as dead tumor cells. (F) The expressions of MHC‐I and PD‐L1 were detected in GL 261 cells transfected with G9a‐shRNA or G9a cDNA. (G) GL261 cells were transfected with vector or G9a cDNA, then co‐cultured with CD8+ T cells at effector: target of 10: 1 in existence of 10 μg/mL anti‐PD‐L1 antibody or not. Cytotoxicity was measured using flow cytometry. *p < 0.05, **p < 0.01 vs. Scr or Vector, #*p* < 0.05 vs. G9a cDNA.

To explore the effect of G9a on local anti‐tumor immunity in tumor tissue, we analyzed subtypes of immune cells in the TME of GL261 transplanting C57BL/6J mice using flow cytometry. G9a knockdown remarkably increased the percentage of CD8+ and CD4+ T cells in tumor tissues. In contrast, G9a overexpression reduced the percentage of these subtypes (Figure 2A). Furthermore, IFN‐γ+ / CD8+ T cells and IFN‐γ+/CD4+ T cells elevated in murine allografts with G9a knockdown and reduced in G9a overexpressing allografts. The examination of PD‐1, a marker of T cell exhaustion, found a decreased expression in CD8+ and CD4+ T cells when tumor cells were transfected with G9a‐shRNA (Figure 2B). Moreover, G9a knockdown significantly decreased the percentages of granulocyte‐like MDSCs (G‐MDSCs) and monocytic MDSCs (M‐MDSCs) (Figure 2C), however, M2‐like macrophages showed no differences (Figure 2D). These results suggested that G9a in glioma cells significantly inhibited local antitumor immune response.

### 3.3 G9a upregulated PD‐L1 expression to inhibit the cytotoxicity of CD8+ T cells

To examine whether the expression of G9a affects the function of effector cells, CD8+ T cells isolated from spleen of C57BL/6J mice were expanded and co‐cultured with GL261 cells. G9a knockdown in GL261 cells increased while G9a overexpression decreased their cytotoxicity by CD8+ T cells (Figure 2E). To explore the mechanism of immunosuppression induced by G9a, we analyzed the expressions of associated molecules in GL261 cells. The decreased PD‐L1 and increased MHC‐I were observed when G9a‐shRNA was transfected into cells. The inverse effects were observed in G9a overexpressing cells (Figure 2F). Then anti‐PD‐L1 monoclonal antibody was applied to block the interaction of PD‐1/PD‐L1. Anti‐PD‐L1 antibody significantly inhibited the decrease in cytotoxicity of CD8+ T cells induced by G9a overexpressing GL261 cells in the co‐culture system (Figure 2G). These results suggested that G9a promoted the formation of immunosuppressive TME through PD‐L1 upregulation.

### 3.4 G9a downregulation suppressed immune activation and promoted cell apoptosis in GSCs

**FIGURE 3:**
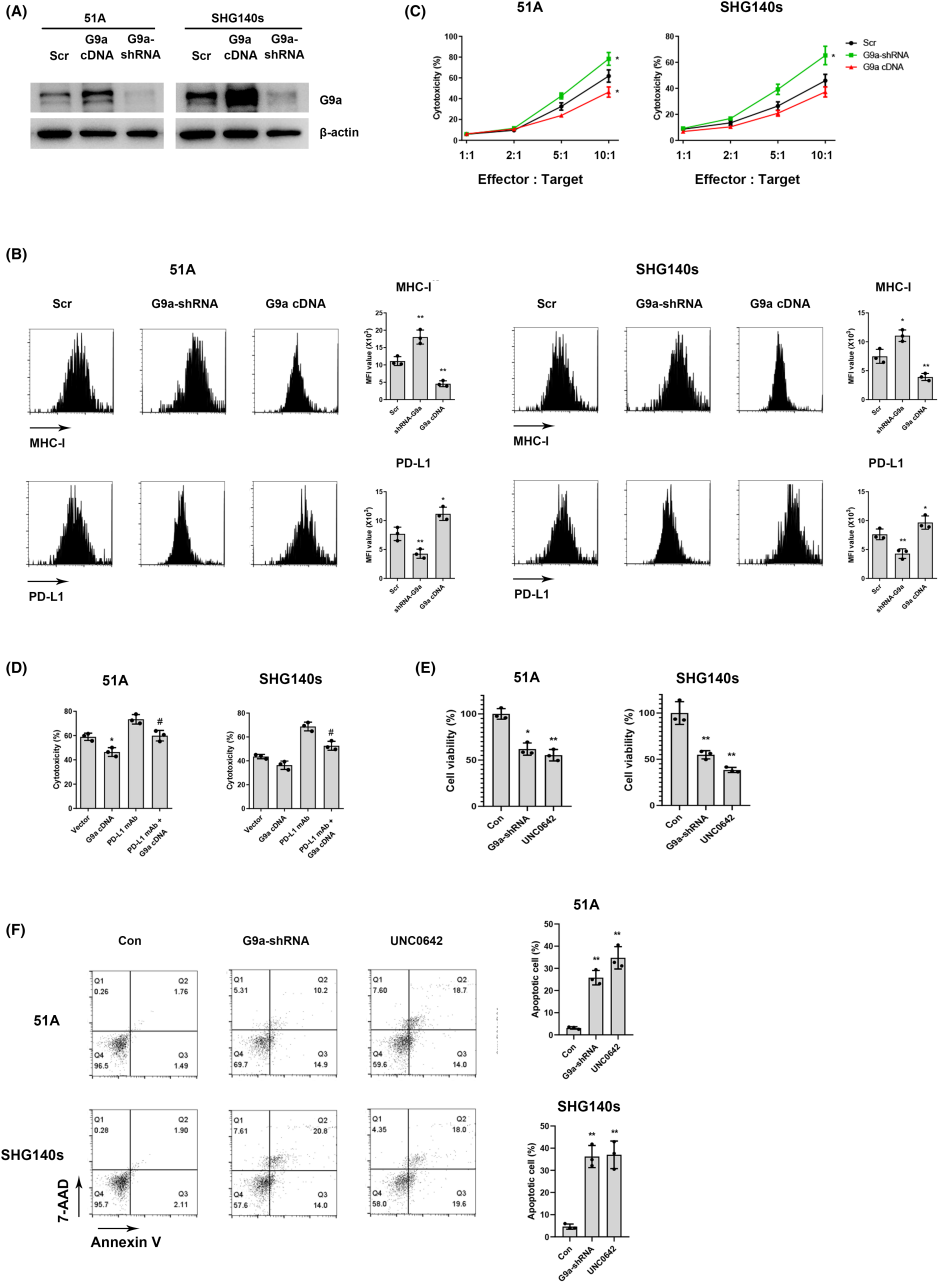
The effect of G9a downregulation on immune activation and cell apoptosis in GSCs. (A) GSCs were transfected with scramble, G9a‐shRNA, and G9a cDNA, and the expressions of G9a were measured using western blot. (B) The expressions of MHC‐I and PD‐L1 were detected using flow cytometry. (C) CD8+ T cells were isolated from healthy human PBMCs, then co‐cultured with GSCs. Cytotoxicity was measured using flow cytometry. (D) GSCs with vector or G9a cDNA co‐cultured with CD8+ T cells in the existence of anti‐PD‐L1 antibody or not, then cytotoxicity was assayed. (E) CCK‐8 assay was performed after GSCs were transfected with G9a‐shRNA or treated with 20 μM UNC0642 for 48 h. (F) Cell apoptosis was detected using flow cytometry after G9a‐shRNA transfection or 20 μM UNC0642 treatment for 24 h. *p < 0.05, **p < 0.01 vs. Scr or Vector, #*p* < 0.05 vs. G9a.

The interaction of GSCs and immune system promotes immune evasion and tumor growth by establishing an immunosuppressive TME.^19^ We investigated the effect of G9a on immune molecules in GSCs. G9a‐shRNA or G9a cDNA transfection into GSCs significantly downregulated or upregulated G9a expression, respectively (Figure 3A). The level of PD‐L1 was decreased in G9a‐shRNA and increased in G9a cDNA‐transfected GSCs compared with scramble, and MHC‐I level was increased in G9a‐shRNA and decreased in G9a cDNA‐transfected GSCs (Figure 3B). G9a‐shRNA transfection in GSCs increased while G9a cDNA decreased their cytotoxicity by CD8+ T cells isolated from human PBMCs (Figure 3C), and anti‐PD‐L1 antibody inhibited the decrease in cytotoxicity of CD8+ T cells in G9a cDNA‐transfected GSCs. (Figure 3D). These data demonstrated that G9a inhibited cytotoxicity of CD8+ T cells via regulating immune molecules expressions in GSCs.

To investigate the effect of G9a on cell proliferation and apoptosis in GSCs, G9a‐shRNA and UNC0642, a G9a inhibitor, were used. UNC0642 treatment showed a dose‐ and time‐dependent decrease in cell proliferation (Figure S1). After G9a knockdown or inhibition in GSCs, CCK8 assays showed that cell proliferation was significantly decreased (Figure 3E). Flow cytometry showed an increased percentage of apoptotic cell (Figure 3F), suggesting the role of G9a on promoting proliferation and inhibiting apoptosis.

### 3.5 G9a downregulation decreased stem characteristics in GSCs

**FIGURE 4:**
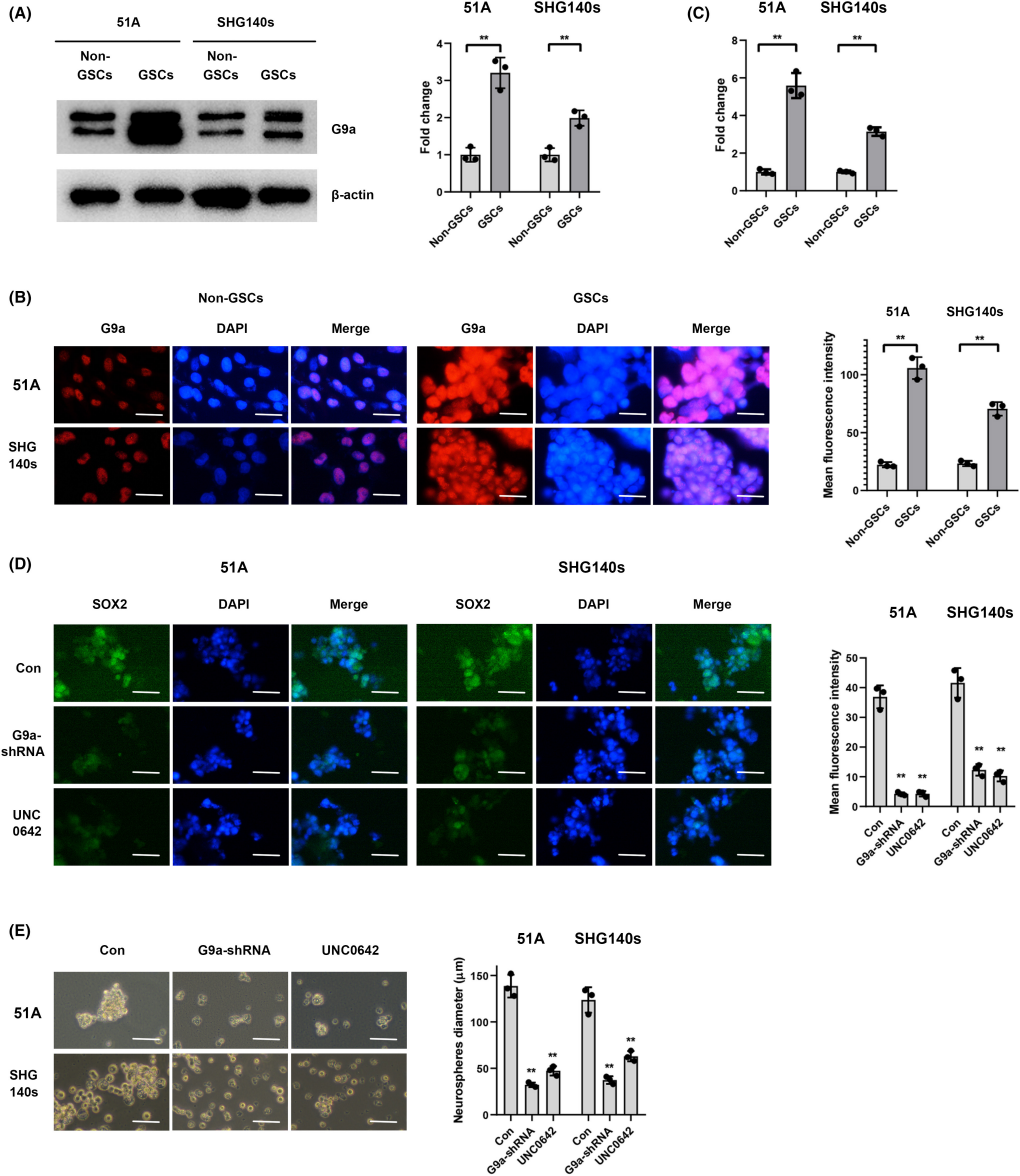
G9a contributes to stemness maintenance of GSCs. (A) G9a protein levels were measured using western blot in non‐GSCs and GSCs. (B) G9a expressions were detected using immunofluorescence staining. Scale bar = 20 μm. (C) Transcriptional levels of G9a were detected using RT‐qPCR. (D) GSCs were transfected with shRNA‐G9a or treated with 20 μM UNC0642 for 24 h, stemness markers were detected using immunofluorescence. Scale bar = 40 μm. (E) Spheroid formation ability was shown after primary spheres were dissociated into single cells Scale bar = 100 μm. *p < 0.05, **p < 0.01.

To investigate the role of G9a in keeping stemness, we first detected the relative levels of G9a in non‐GSCs and GSCs. Western blot (Figure 4A and Figure S2A), immunofluorescence (Figure 4B and Figure S2B), and RT‐qPCR (Figure 4C and Figure S2C) demonstrated that G9a expressions were increased in identified 4 GSC lines than in corresponding non‐GSC lines. G9a knockdown or inhibition dramatically decreased the expression of pluripotent transcription factor SOX2 by immunofluorescence assay (Figure 4D) and spheroid formation ability (Figure 4E) in GSCs.

### 3.6 G9a promoted stemness characteristics of GSCs and suppressed immune response via Notch signaling pathway

**FIGURE 5:**
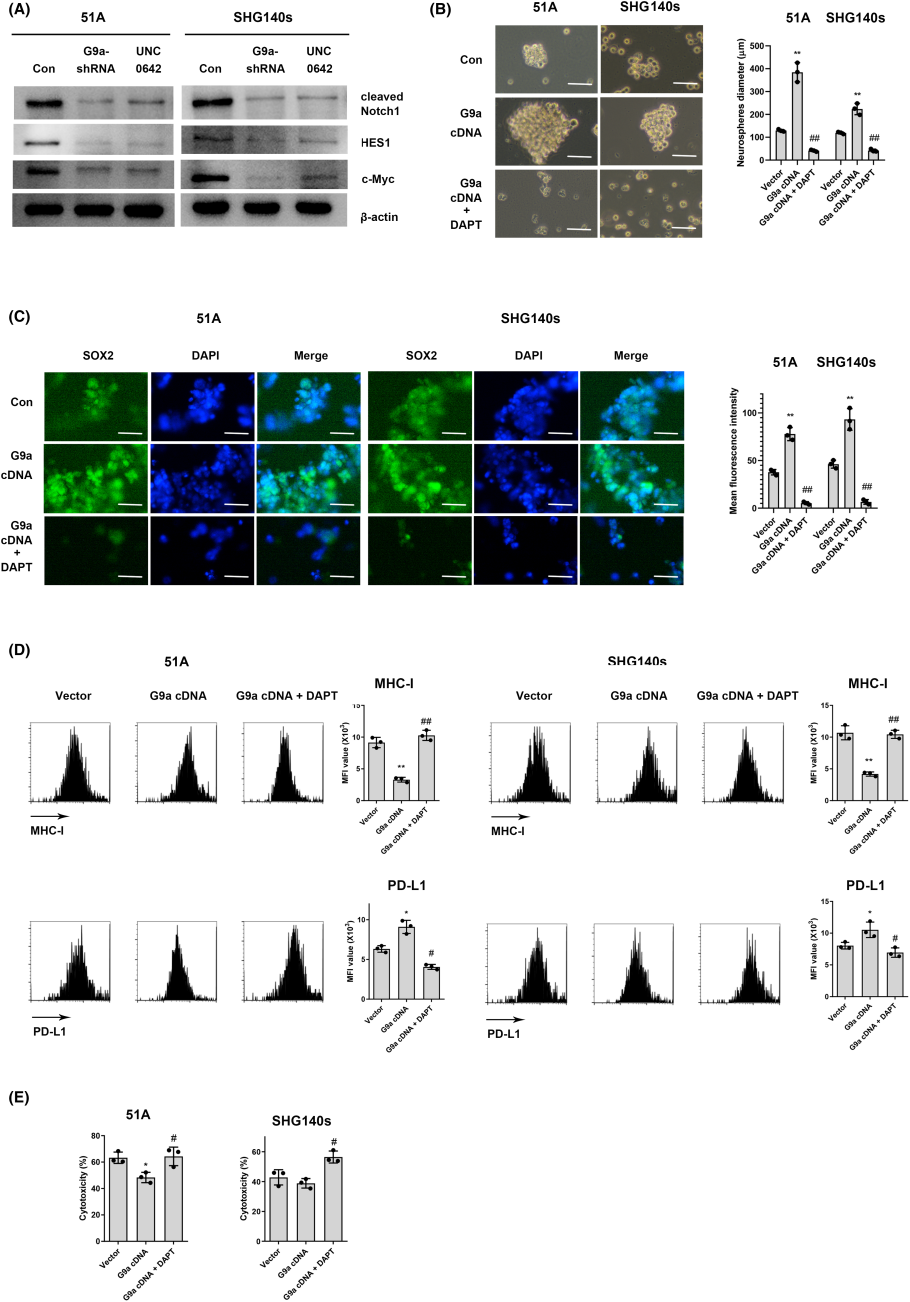
G9a activates Notch1 signaling pathway to maintain stemness in GSCs. (A) The expressions of Notch1 signaling and its downstream target genes were detected using western blot after G9a‐shRNA transfection or UNC0642 treatment in GSCs. GSCs were treated with 10 μM DAPT, a Notch1 signaling pathway inhibitor, for 12 h, then spheroid formation (B), the expressions of stemness markers using immunofluorescence assay (C) and the expressions of immune molecules (D) were observed in G9a cDNA‐transfected GSCs. Scale bars are 100 μm in B and 40 μm in C. (E) Cytotoxicity of CD8+ T cells was assayed after co‐culture with GSCs. *p < 0.05, **p < 0.01 vs. vector; #*p* < 0.05, ##*p* < 0.01 vs. G9a cDNA.

Notch1, an oncogene in GBM, is required for proliferation and survival of stem cells, and Notch signaling plays an important role in immune responses in tumors.^20^ To investigate whether G9a regulates immune response through Notch1 signaling pathway, we observed the expressions of Notch1 pathway‐associated genes after G9a knockdown or inhibition in GSCs. The expressions of Notch 1 signaling and its downstream target genes HES1 were significantly increased in GSCs than in non‐GSCs (Figure S3). Compared with control group, G9a knockdown or inhibition decreased expressions of Notch1, HES1, and c‐myc (Figure 5A). Furthermore, we transfected G9a cDNA to observe the effect of G9a on cell stemness and immune molecules regulation when Notch1 signaling pathway was blocked. Notch1 was activated to maintain stemness in GSCs (Figure S3), and Notch1 pathway blockade abrogated the increase in spheroid formation ability (Figure 5B) and the expressions of stemness markers (Figure 5C) induced by G9a overexpression. Moreover, the expressions of MHC‐I and PD‐L1 were similarly reverted by Notch1 inhibitor (Figure 5D). In addition, Notch1 inhibitor increased cytotoxicity of CD8+ T cells in co‐culture with G9a overexpressing GSCs (Figure 5E). These data suggested that G9a maintained stemness characteristics of GSCs through Notch1 signaling pathway.

### 3.7 G9a activated Notch signaling via binding to Fbxw7 promotor

**FIGURE 6:**
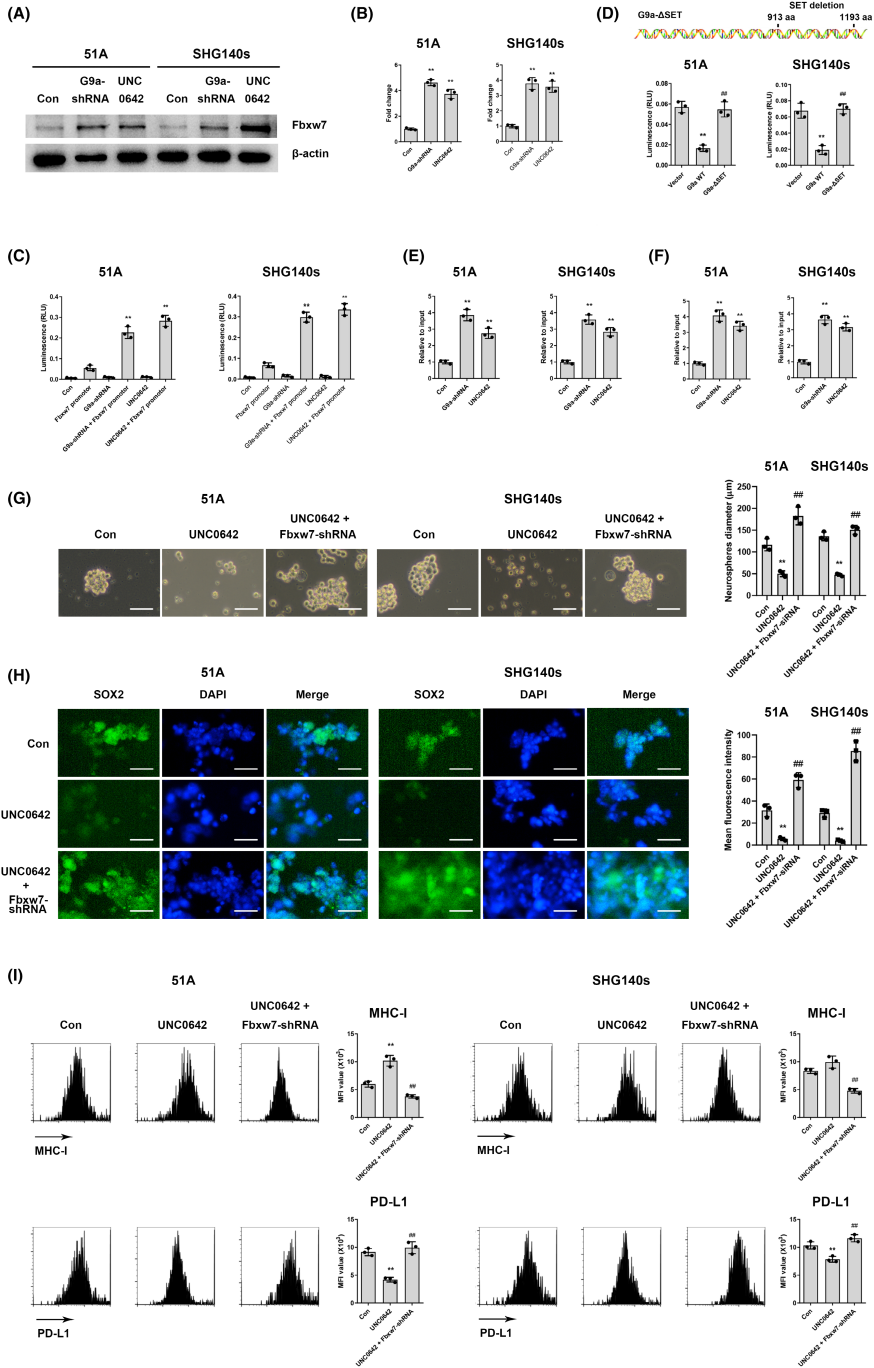
G9a upregulates Notch signaling by suppressing Fbxw7 promotor H3K9me2. Fbxw7 protein level using western blot (A) and mRNA level using RT‐qPCR (B) were detected in G9a‐shRNA transfected or UNC0642‐treated GSCs. **p < 0.01 vs. control. (C) Transcriptional activity was measured using dual‐luciferase reporter assays in G9a‐shRNA transfected or UNC0642‐treated GSCs after plasmid with Fbxw7 promoter truncation was transfected into cells for 24 h. **p < 0.01 vs. Fbxw7 promoter. (D) Plasmids carrying in combination of Fbxw7 promoter truncation and G9a WT or G9a‐ΔSET sequence were transfected into GSCs, luciferase activity was detected using dual‐luciferase reporter assay. **p < 0.01 vs. vector; ##*p* < 0.01 vs. G9a WT. ChIP assay was performed using G9a (E) or H3K9me2 (F) antibody. **p < 0.01 vs. control. (G) After UNC0642 treatment, spheroid formation (G), the expressions of stemness markers (H), and immune molecules (I) were measured in Fbxw7‐shRNA‐transfected GSCs. Scale bars are 100 μm in G and 40 μm in H. *p < 0.05; **p < 0.01 vs. control; ##*p* < 0.01 vs. UNC0642.

Fbxw7 suppresses cancer niche proliferation through ubiquitination and degradation of Notch pathway.^21^ To investigate the mechanism of Fbxw7 on regulating stemness in GSCs, we speculated that Fbxw7 might be a potential G9a interaction partner. Both western blot (Figure 6A) and RT‐qPCR (Figure 6B) results showed that the expressions of Fbxw7 were increased by G9a knockdown or inhibition, suggesting that G9a regulated Fbxw7 expression in transcriptional level. Furthermore, recombinant pGL3 plasmid carrying Fbxw7 promotor was constructed and transfected into GSCs. Dual‐luciferase assays showed that luciferase expression was notably increased by G9a knockdown or inhibition (Figure 6C). To examine whether G9a regulates Fbxw7 transcription through the methyltransferase activity, we transfected overexpressing plasmids carrying a sequence of wild‐type G9a (G9A WT) or SET domain‐deleted G9a (G9a‐ΔSET), resulting in loss of methyltransferase activity.^18^ G9a WT rather than G9A‐ΔSET increased luciferase activity (Figure 6D), suggesting that G9a methyltransferase activity was important for Fbxw7 transcriptional regulation. ChIP assay found that G9a knockdown increased the input of Fbxw7 promotor (Figure 6E), meanwhile, ChIP assay using H3K9me2 antibody showed consistent results (Figure 6F), suggesting that G9a directly bound to Fbxw7 promotor and regulated Fbxw7 promotor activity via inducing H3K9me2. Moreover, Fbxw7 knockdown reversed the decrease in spheroid formation ability (Figure 6G) and SOX2 expression (Figure 6H), the increase in MHC‐I and the decrease in PD‐L1 expressions mediated by G9a inhibition (Figure 6I). Taken together, these data indicated that G9a upregulated Notch signaling by suppressing Fbxw7 promotor H3K9me2.

## 4 DISCUSSION

In this study, we revealed that G9a positively regulated immunosuppression formation in a mouse model. G9a downregulated the expression of MHC‐I, presenting intracellular antigens, and upregulated the expression of PD‐L1, an immune checkpoint molecule, on the surface of tumor cells through Notch pathway. Mechanistically, G9a promoted the activation of Notch signaling by inhibiting the expression of Fbxw7. G9a directly facilitated the histone methylation of Fbxw7 promoter to suppress gene transcription. Our results indicated that targeting G9a might be a potential strategy for GSCs elimination and GBM therapy by decreasing stemness and remodeling immune microenvironment.

We injected tumor cells with luciferase lentivirus into mice and measured the size of intracranial tumors by imaging analysis in this study. Several excellent imaging modalities have been developed recently for brain tumor studies, such as quantifying millions of microvessels and their distribution,^22^ conjugating anti‐ALCAM antibodies to microparticles of iron oxide as a contrast agent^23^ and single‐dose dual‐echo acquisitions protocol,^24^ which promoted imaging development in tumor localization and boundary outline.

G9a performed important function in human GBM cells. In previous studies, individual G9a inhibitor BIX01294 at low concentrations showed a moderate effect on the viability of U87 cells, and a synergistic effect was identified on inhibition of glioma cell viability in combination with G9a, EZH2, and HDAC inhibitors.^13^ BIX01294 is effective radiosensitizers of human glioma cells, and loss of H3K9 methylation reduces H3K9‐dependent DNA damage and inhibits DNA double‐strand breaks repair.^14^ Different results showed that BIX01294 stimulated the sphere formation rate of GSCs, and upregulated SOX2 and CD133 expressions.^25^ Another study demonstrated that G9a ectopic overexpression did not affect the expression of SOX2 in GBM cell lines and an ER(−) breast cancer cell line. However, consistent data appeared in two ER(+) breast cancer cell lines, and chemical G9a inhibitor decreased SOX2 protein levels. Cell migration, invasion, and mammosphere formation were correlated with G9a expression. BIX01294 treatment in mouse embryonic stem cells resulted in reduction in SOX2 protein expression but increase in transcriptional level.^26^

Some evidences showed that G9a bound key gene to change cell biological characteristics. G9a directly bound to the HIF‐1α and catalyzed lysine 674 methylation of HIF‐1α, suppressing HIF‐1 transcriptional activity and downstream target genes expressions in U251MG cells, which decreased HIF‐1‐dependent migration under hypoxia.^27^ G9a bound to the promoters of autophagy‐related genes including LC3B and WIPI1 and differentiation‐related genes including GFAP and TUBB3 in GSCs, and autophagy inhibitor decreased the expressions of GFAP and TUBB3 in BIX01294‐treated cells.^15^

Accumulating evidence showed that various factors such as CXCL1^28^ and GDF15^29^ were associated with malignant progression, immune microenvironment, and conventional therapy resistance. The interactions of Notch signaling and the immune response were also demonstrated in tumors.^20^ Notch signaling promoted macrophage polarization toward M2 phenotype.^30^ Aberrant expression of the co‐stimulatory receptor Notch1 in CD4+ T cells was associated with the failure of the PD‐1/PD‐L1 immune checkpoint.^31^ Our data showed that G9a upregulated PD‐L1 and downregulated MHC‐I expressions to inhibit immune response by Notch activation in GSCs. Consistent with our results, loss of Notch activity impaired MHC‐I and cytokine expressions, reduced the ratio of anti‐tumor immune cell populations, and increased immunosuppressive tumor‐associated macrophages. T cells depletion in a mouse glioma model promoted the effect of Notch inhibition and favored tumor initiation.^10^ PD‐L1 expression on breast cancer stem cells was partly dependent on Notch via PI3K/AKT pathway, and Notch3 as a mediator for PD‐L1 was important for maintaining stemness.^32^

Fbxw7 mainly serves as a cancer suppressor, and mutation or Loss of Fbxw7 is frequently found in multiple human tumors including GBM.^33^ As a component of ubiquitin ligase complexes, Fbxw7 exerts its important function through ubiquitin‐mediated degradation of its substrates. Notch1 pathway as a substrate of Fbxw7 is regulated by Fbxw7‐dependent ubiquitination and proteolysis, and downregulation of Fbxw7 results in the accumulation of target proteins.^21^ Loss of Fbxw7 leads to the increase in Notch signaling by prolonging NICD half‐life, which abrogates ubiquitylation and proteasome‐mediated destruction.^34^ Given to the key roles of Fbxw7 on Notch ubiquitylation, we investigated Fbxw7 regulation by G9a in this research, and the results showed G9a directly facilitated the histone methylation of Fbxw7 gene promoter to suppress Fbxw7 transcription, suggesting that G9a upregulated Notch1 signaling by histone methylation on Fbxw7 promotor.

## AUTHOR CONTRIBUTIONS

CY wrote the original draft. CY, LB, and CL performed the experiments and analyzed the data. CY performed gene transfection, flow cytometry, immunofluorescence, western blot, RT‐qPCR, and ChIP assay. CY, LB, and CL established animal model. LY isolated and cultured CD8+ T cells and performed cytotoxicity assay. SX edited the manuscript. YH and YZ supervised the project. WY and ST designed the experiments, acquired funding, supervised the project, and performed review and editing.

## CONFLICT OF INTEREST STATEMENT

The authors declare no conflict of interest.

## Supporting information


Figure S1.
Figure S2.
Figure S3.

## Data Availability

The authors declare that all relevant data of this study are available within the article or from the corresponding author on reasonable request.
