# Supplementary material for: G9a promotes immune suppression by targeting the Fbxw7/Notch pathway in glioma stem cells
Source: CNS Neurosci Ther. 2023 Mar 27;29(9):2508–21. doi: 10.1111/cns.14191 (PMC10401078; doi:10.1111/cns.14191)
Supplement: Supplementary file 1 — Figure S1. Figure S2. Figure S3. [file CNS-29-2508-s001.docx]

**Supplementary Figures**


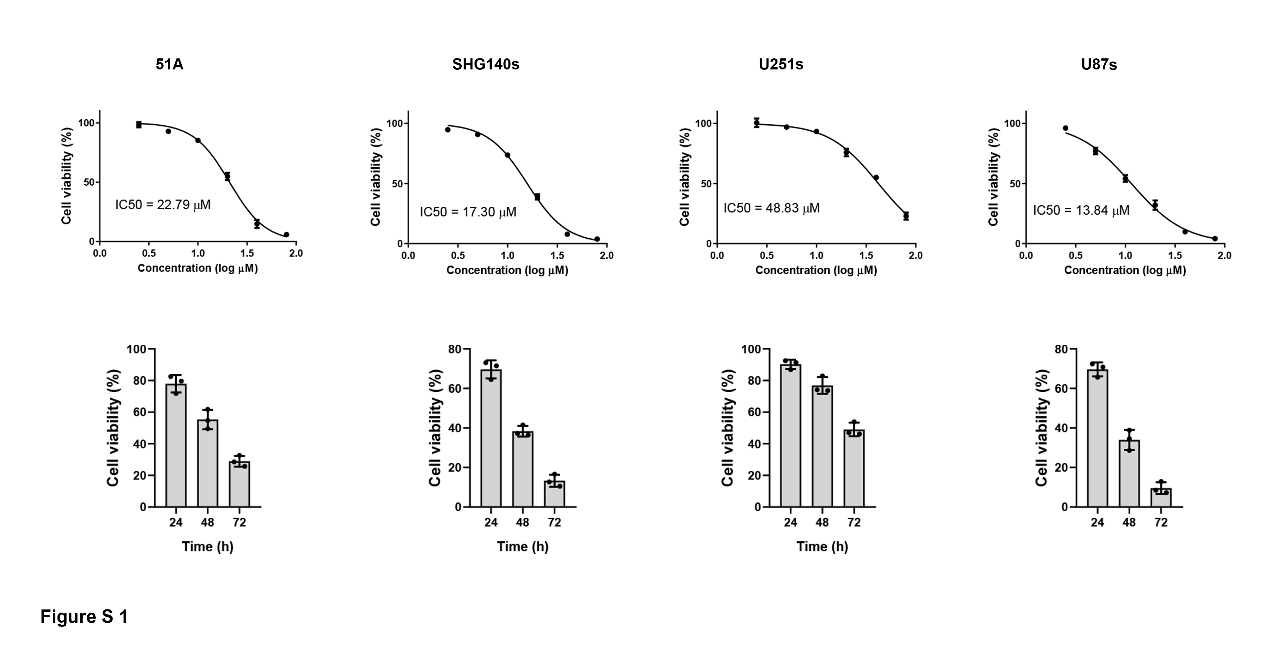


**Supplementary Fig. S1** **Cell proliferation was showed after UNC0642 treatment.** GSCs were treated with UNC0642 in indicated time point for different dosage, cell proliferation was detected using CCK-8 assay. *P<0.05, ***P* < 0.01.


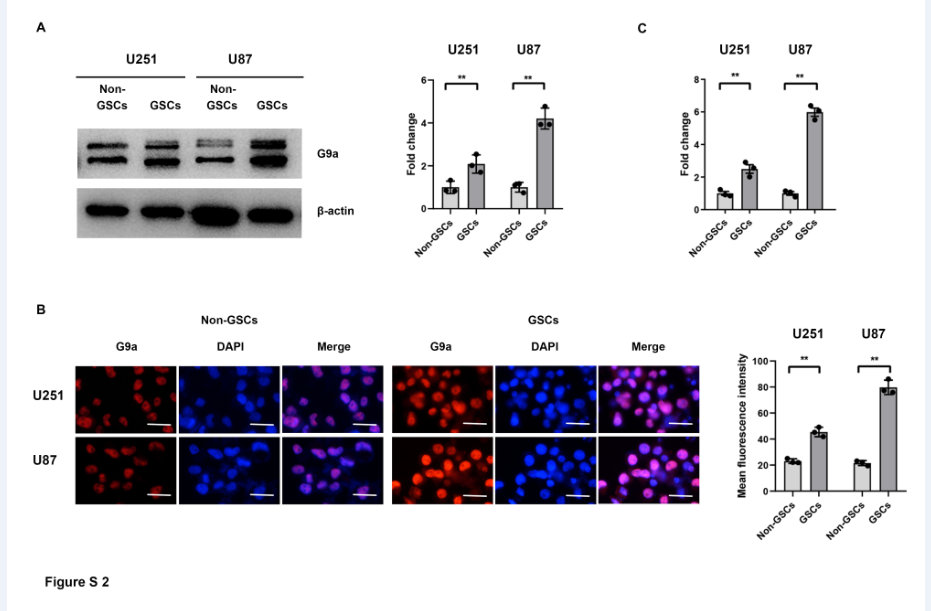


**Supplementary Fig. S2 G9a is upregulated in GSCs compared with non-GSCs.** G9a protein levels using Western blot (A) and immunofluorescence staining (B) and mRNA level using RT-qPCR were measured. Scale bar = 20 μm. **P* < 0.05, ***P* < 0.01.


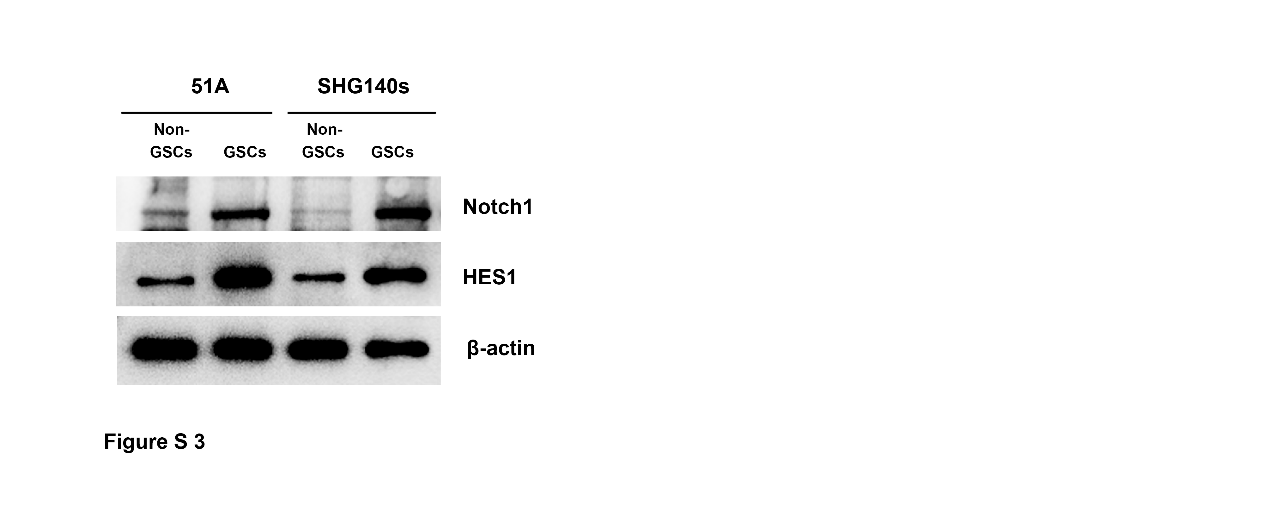


**Supplementary Fig. S2 Notch pathway associated genes expressions in GSCs and non-GSCs.**
